# Gain-of-Function Research: Ethical Analysis

**DOI:** 10.1007/s11948-016-9810-1

**Published:** 2016-08-08

**Authors:** Michael J. Selgelid

**Affiliations:** Centre for Human Bioethics, Monash University, Melbourne, Australia

**Keywords:** Gain-of-function research, Dual-use research, Biosafety, Biosecurity, Risk-benefit assessment, Decision theory

## Abstract

Gain-of-function (GOF) research involves experimentation that aims or is expected to (and/or, perhaps, actually does) increase the transmissibility and/or virulence of pathogens. Such research, when conducted by responsible scientists, usually aims to improve understanding of disease causing agents, their interaction with human hosts, and/or their potential to cause pandemics. The ultimate objective of such research is to better inform public health and preparedness efforts and/or development of medical countermeasures. Despite these important potential benefits, GOF research (GOFR) can pose risks regarding biosecurity and biosafety. In 2014 the administration of US President Barack Obama called for a “pause” on funding (and relevant research with existing US Government funding) of GOF experiments involving influenza, SARS, and MERS viruses in particular. With announcement of this pause, the US Government launched a “deliberative process” regarding risks and benefits of GOFR to inform future funding decisions—and the US National Science Advisory Board for Biosecurity (NSABB) was tasked with making recommendations to the US Government on this matter. As part of this deliberative process the National Institutes of Health commissioned this Ethical Analysis White Paper, requesting that it provide (1) review and summary of ethical literature on GOFR, (2) identification and analysis of existing ethical and decision-making frameworks relevant to (i) the evaluation of risks and benefits of GOFR, (ii) decision-making about the conduct of GOF studies, and (iii) the development of US policy regarding GOFR (especially with respect to funding of GOFR), and (3) development of an ethical and decision-making framework that may be considered by NSABB when analyzing information provided by GOFR risk-benefit assessment, and when crafting its final recommendations (especially regarding policy decisions about funding of GOFR in particular). The ethical and decision-making framework ultimately developed is based on the idea that there are numerous ethically relevant dimensions upon which any given case of GOFR can fare better or worse (as opposed to there being necessary conditions that are either satisfied or not satisfied, where all must be satisfied in order for a given case of GOFR to be considered ethically acceptable): research imperative, proportionality, minimization of risks, manageability of risks, justice, good governance (i.e., democracy), evidence, and international outlook and engagement. Rather than drawing a sharp bright line between GOFR studies that are ethically acceptable and those that are ethically unacceptable, this framework is designed to indicate where any given study would fall on an ethical spectrum—where imaginable cases of GOFR might range from those that are most ethically acceptable (perhaps even ethically praiseworthy or ethically obligatory), at one end of the spectrum, to those that are most ethically problematic or unacceptable (and thus should not be funded, or conducted), at the other. The aim should be that any GOFR pursued (and/or funded) should be as far as possible towards the former end of the spectrum.

## Executive Summary

Gain-of-function (GOF) research involves experimentation that aims or is expected to (and/or, perhaps, actually does) increase the transmissibility and/or virulence of pathogens. Such research, when conducted by responsible scientists, usually aims to improve understanding of disease causing agents, their interaction with human hosts, and/or their potential to cause pandemics. The ultimate objective of such research is to better inform public health and preparedness efforts and/or development of medical countermeasures. Despite these important potential benefits, GOF research (GOFR) can pose risks regarding biosecurity and biosafety. GOFR is a subset of “dual-use research”—i.e., research that can be used for both beneficial and malevolent purposes. Whereas the dual-use life science research debate has largely focused on biosecurity dangers associated with potential malevolent use of research, the GOFR debate has more explicitly focused on risks involving both biosecurity and biosafety—the point being that creation of especially dangerous pathogens might pose highly significant biosafety risks that are independent of, and perhaps more feasible to measure/assess than, risks associated with malevolent use.

Following controversy surrounding research, published in 2012, that led to the creation of highly pathogenic H5N1 (avian) influenza virus strains that were airborne transmissible between ferrets—and more recent reports of biosafety mishaps involving anthrax, smallpox, and H5N1 in government laboratories—in 2014 the administration of US President Barack Obama called for a “pause” on funding (and relevant research with existing US Government funding) of GOF experiments involving influenza, SARS, and MERS viruses in particular. This pause applies specifically to experiments that “may be reasonably anticipated to confer attributes … such that the virus would have enhanced pathogenicity and/or transmissibility in mammals via the respiratory route” (White House 2014). With announcement of this pause, the US Government launched a “deliberative process … to address key questions about the risks and benefits of gain-of-function studies” (White House 2014) to inform future funding decisions—and the National Science Advisory Board for Biosecurity (NSABB) was tasked with making recommendations to the US Government on this matter. As part of this deliberative process, the National Institutes of Health (NIH) commissioned this Ethical Analysis White Paper providing:

1. Review and summary of ethical literature on GOFR;
2. Identification and analysis of existing ethical and decision-making frameworks relevant to (i) the evaluation of risks and benefits of GOFR, (ii) decision-making about the conduct of GOF studies, and (iii) the development of US policy regarding GOFR (especially with respect to funding of GOFR); and
3. Development of an ethical and decision-making framework that may be considered by NSABB when analyzing information provided by GOFR risk-benefit assessment, and when crafting its final recommendations (especially regarding GOFR funding policy decisions in particular).

The ethical literature (discussed below) on GOFR to date has primarily focused on

- Biosafety concerns—e.g., that a devastating pandemic could potentially result from a laboratory accident involving an especially dangerous pathogen created via GOFR
- The need for objective risk-benefit analysis, broader community engagement/consultation, and more transparent GOFR decision- and policy-making
- The need to minimize risks—and controversy surrounding the nature and magnitude of likely risks of GOFR
- The requirement that research benefits outweigh risks—and controversy surrounding the nature and magnitude of likely benefits of GOFR

Following (1) discussion of the limitations of risk-benefit assessment as a guide to decision- and policy-making and (2) identification of numerous existing ethical and decision-making frameworks, and analysis of their general strengths and weaknesses and/or specific applicability to GOFR, this White Paper ultimately develops/proposes a framework for GOFR decision-and policy-making (especially regarding funding of GOFR) comprised of the following principles:

1. *Research Imperative* The ethical acceptability of GOFR posing extraordinary risks partly depends on the importance of the research question it aims to address.
2. *Proportionality* The ethical acceptability of extraordinarily risky GOFR partly depends on the extent to which there is reasonable expectation that the research in question will (1) yield answers to the target public health question and (2) ultimately result in benefits that outweigh risks involved.
3. *Minimization of Risks* Other things being equal, the ethical acceptability of a GOFR study is a function of the degree to which (1) there is confidence that no less risky forms of research would be equally beneficial and (2) reasonable steps have been made to minimize risks of the GOFR study in question.
4. *Manageability of Risks* Other things being equal, the more manageable the risks of a GOFR study, the more ethically acceptable the study would be. Conversely, the more important/beneficial a GOFR study is expected to be, the more we should be willing to accept potentially unmanageable risks.
5. *Justice* Because justice requires fair sharing of benefits and burdens, the ethical acceptability of GOFR partly depends on the degree to which (1) risks fall on some people more than others, (2) risks fall on those who are unlikely to benefit, and/or (3) any resulting harms are uncompensated.
6. *Good Governance—Democracy* GOFR decision- and policy-making should (insofar as possible) reflect the ultimate values, value weightings, and risk-taking strategies of public citizens.
7. *Evidence* Decision- and policy-making regarding GOFR should be based on more/better evidence regarding risks, benefits, (means of) risk minimization, who is likely to benefit or be harmed by research, and the values, value weightings, and risk-taking strategies of public citizens.
8. *International Outlook and Engagement* Because risks and benefits of GOFR (can) affect the global community at large, the ethical acceptability of GOFR partly depends on the extent to which it is accepted internationally. Decision- and policy-making regarding GOFR should (insofar as possible) involve consultation, negotiation, coordination, and related forms of active engagement with other countries.

This framework is based on the idea that there are numerous ethically relevant dimensions upon which any given case of GOFR can fare better or worse (as opposed to there being necessary conditions that are either satisfied or not satisfied, where all must be satisfied in order for a given case of GOFR to be considered ethically acceptable). Rather than drawing a sharp bright line between GOFR studies that are ethically acceptable and those that are ethically unacceptable, this framework is designed to indicate where any given study would fall on an ethical spectrum—where imaginable cases of GOFR might range from those that are most ethically acceptable (perhaps even ethically praiseworthy or ethically obligatory) (i.e., those that fare best with respect to all 8 dimensions), at one end of the spectrum, to those that are most ethically problematic or unacceptable (i.e., those that fare worst regarding all 8 dimensions, and thus clearly should not be funded/conducted), at the other. The aim should be that any GOFR pursued (and/or funded) should be as far as possible towards the former end of the spectrum.

## Introduction

Gain-of-function (GOF) research involves experimentation that aims or is expected to (and/or, perhaps, actually does) increase the transmissibility and/or virulence of pathogens. Such research, when conducted by responsible scientists, usually aims to improve understanding of disease causing agents, their interaction with human hosts, and/or their potential to cause pandemics. The ultimate objective of such research is to better inform public health and preparedness efforts and/or development of medical countermeasures. Despite these important potential benefits, GOF research (GOFR) can pose risks regarding biosecurity and biosafety.

GOFR is a subset of “dual-use research”—i.e., research that can be used for both beneficial and malevolent purposes (Miller and Selgelid 2008; National Research Council 2004). ‘Dual-use research of concern’ (DURC) refers to dual-use research for which the consequences of malevolent use would be exceptionally severe (whereas almost any research might be considered “dual-use” broadly conceived—because almost any research, or just about anything for that matter, can be used for some malevolent purpose or other). Of particular concern in the context of life science research is that advances in biotechnology may enable development and use of a new generation of biological weapons of mass destruction.

DURC has thus been one of the most hotly debated science policy issues during the 21st century, with controversy surrounding a series of published experiments with potential implications for biological weapons-making. Such studies include the genetic engineering of a superstrain of the mousepox virus in 2001 (Jackson et al. 2001), the artificial synthesis (via synthetic genomics) of a “live” polio virus from chemical components in 2002 (Cello et al. 2002), and the reconstruction (via synthetic genomics) of the 1918 “Spanish Flu” virus in 2005 (Tumpey et al. 2005). Though all of these studies involved legitimate aims, critics argued that they should not have been conducted and/or published. Some argued that publishing studies like these in full detail provided “recipes” for especially dangerous potential biological weapons agents to would-be bioterrorists. Many who acknowledged such potential dangers, on the other hand, argued that benefits of publication outweighed risks involved.

The most controversial dual-use life science experiments to date involved the creation of highly pathogenic H5N1 (avian) influenza virus strains that were airborne transmissible between ferrets, which provide the best model for influenza in humans (Herfst et al. 2012; Imai et al. 2012). This research addressed an important scientific question—i.e., Might it be possible for H5N1 to naturally evolve into a human-to-human transmissible strain and thus result in a pandemic?—and (purportedly) yielded an affirmative answer. After the US National Science Advisory Board for Biosecurity (NSABB) initially recommended that these studies should be published in a redacted form (i.e., including key findings, while omitting detailed description of materials and methods), it later approved publication of revised versions in full, and the papers were published in 2012. Advocates of these studies/publications argued that they would improve surveillance of H5N1 in nature (facilitating early identification of, and thus better response to, the emergence of potential pandemic strains) and facilitate development of vaccines that might be needed to protect against pandemic strains of the virus. Critics questioned the validity of claims about such benefits and argued that the studies might facilitate creation of biological weapons agents that could kill millions, or possibly even billions, of people.

While the concern about the biological weapons implications of this ferret H5N1 research pertains to dangers of dual-use life science research as traditionally conceived, many of the objections to this research additionally addressed the danger that the pathogens created might have escaped from laboratories, and potential consequences thereof—and there were particular concerns about the conditions under which this research was conducted (e.g., the safety level of the laboratories where this research was conducted). Controversy surrounding these ferret H5N1 experiments has thus lead to a significant shift in debate about dual-use research to framing in terms of “gain-of-function research”. Whereas the dual-use debate largely focused on biosecurity dangers associated with potential malevolent use of research, the GOFR debate has more explicitly focused on risks involving *both biosecurity and biosafety*—the point being that creation of especially dangerous pathogens might pose highly significant biosafety risks that are independent of, and perhaps more feasible to measure/assess than, risks associated with malevolent use.

Since the first high-profile DURC life science experiments were published in the early 2000s, much policy debate has surrounded questions about how DURC should be governed. Among other things, it has been argued that increased oversight of research and/or publication of potentially dangerous discoveries may be necessary, that codes of conduct for scientists (explicitly addressing dual use issues) should be adopted, and/or that scientists should be further educated about the dual use phenomenon and ethics; and relevant policies have been implemented to varying degrees in different countries. In light of the ferret H5N1 research controversy, furthermore, influenza researchers imposed a voluntary moratorium on GOF studies from January 2012 to February 2013; and the US Government developed/adopted policy regarding the funding of GOF H5N1 studies in 2013 (Department of Health and Human Services 2013).

Following more recent reports of biosafety mishaps involving anthrax, smallpox, and H5N1 in government laboratories—and burgeoning debate regarding biosafety risks of GOFR more generally (Kaiser 2014)—in 2014 the administration of US President Barack Obama called for a “pause” on funding (and relevant research with existing US Government funding) of GOF experiments involving influenza, SARS, and MERS viruses in particular. This pause applies specifically to experiments that “may be reasonably anticipated to confer attributes … such that the virus would have enhanced pathogenicity and/or transmissibility in mammals via the respiratory route” (White House 2014). With announcement of this pause, the US Government launched a “deliberative process … to address key questions about the risks and benefits of gain-of-function studies” (White House 2014) to inform future funding decisions—and NSABB was tasked with making recommendations to the US Government on this matter. As part of this deliberative process, the National Institutes of Health (NIH) commissioned this Ethical Analysis White Paper providing:

1. Review and summary of ethical literature on GOFR;
2. Identification and analysis of existing ethical and decision-making frameworks relevant to (i) the evaluation of risks and benefits of GOFR, (ii) decision-making about the conduct of GOF studies, and (iii) the development of US policy regarding GOFR (especially with respect to funding of GOFR); and
3. Development of an ethical and decision-making framework that may be considered by NSABB when analyzing information provided by GOFR risk-benefit assessment, and when crafting its final recommendations (especially regarding GOFR funding policy decisions in particular).

## Gain-of-Function Research Ethics: State of Debate

Just as (bio)ethicists were slow to engage in debate about dual-use life science research more generally (Selgelid 2010), it is noteworthy that (with very few exceptions) most of the existing literature explicitly addressing gain-of-function research (i.e., using the language of ‘gain-of-function research’) has not been authored by (bio)ethicists in particular. Even when authored by scholars from other disciplines, furthermore, most of the existing ethically relevant GOFR literature is neither explicitly focused on ethics (e.g., using the language of ‘ethics’ in titles, abstracts, or key words) nor published in (bio)ethics journals. On the other hand, much of the literature surrounding GOFR controversy is (largely) implicitly concerned with ethics (whether or not the language of ‘ethics’ is front and center) insofar as normative considerations, values, questions about how to weigh risks/harms against benefits, and questions about “what ought to be done”—all of which fall squarely within the domain of ethics—are of central concern. The following thus aims to summarize the main ethical issues/points raised in literature explicitly concerned with GOFR (using the language of ‘gain-of-function research’) whether or not the papers in question were authored by ethicists, published in ethics journals, or explicitly employ widespread use of the language of ‘ethics’.^1^

### Biocontainment

A distinct aspect of the shift in debate from framing in terms of “dual-use research” to “gain-of-function research” has been focus on biosafety concerns—e.g., that a devastating pandemic could potentially result from a laboratory accident involving an especially dangerous pathogen created via GOFR. In light of Ron Fouchier’s claim that the ferret-transmissible strain of H5N1 he produced is “probably one of the most dangerous viruses you can make” (Enserink 2011) and (previous) NSABB chair Paul Keim’s claim that “I can’t think of another pathogenic organism as scary as this one [created by Fouchier’s team] … I don’t think anthrax is scary at all compared to this” (Enserink 2011), for example, some critics argued that the study in question should have been, and/or that future similar research should be, conducted in laboratories with the highest bio-containment level—i.e., biosafety level 4 (BSL-4), as opposed to BSL-3 (“enhanced”) in which this research was done (Swazo 2013). Fouchier has, in response, pointed out that his research received necessary institutional biosafety review/approval; and others have argued that his research (given employment of safety measures beyond ordinary BSL-3, including vaccination of lab workers against H5N1) in effect involved safety equivalent to BSL-4 (Roos 2012). Anthony Fauci (Director of the US National Institute of Allergy and Infectious Diseases) has concluded that “the scientists who triggered this debate [including Fouchier] … have conducted their research properly and under the safest and most secure conditions” (Fauci 2012, p. 1).

Additional biosafety concerns involve potential dangers associated with proliferation of GOFR, which is arguably likely to occur as more similar work is conducted/published. Whether or not GOFR has been adequately safe to date, similar future research might be conducted in suboptimal conditions—e.g., in countries/institutions with weaker infrastructure and/or research oversight systems (Evans et al. 2015; Fauci 2012; Gronvall 2014; Lipsitch and Galvani 2014; Wain-Hobson 2014). Part of the resistance to insistence that additional similar research be conducted in BSL-4, on the other hand, is that this might unnecessarily increase expense, reduce efficiency, and/or inequitably deem relevant research impermissible in less wealthy countries (Lipkin 2012).

### Broad Community Engagement, Risk-Benefit Analysis, and Transparency

One clear consensus in (ethically relevant) literature addressing GOFR is that there is need for broader community engagement/consultation and more transparent decision- and policy-making (Duprex et al. 2015; Evans et al. 2015; Fauci 2012; Imperiale and Casadevall 2015; Lipsitch and Galvani 2014; Lipsitch and Inglesby 2014; Pfeiffer 2015; Suk et al. 2014). Part of the concern here, hopefully to be addressed by the deliberative process initiated by the US Government, is the perception (at least in the eyes of some) that much of the relevant debate and/or decision-making to date has been dominated by a limited subset of the scientific community and/or by people or parties with potential conflicts of interest. Because the potential risks and benefits of GOFR affect the public at large, it has been argued that more public input to debate and decision-making is necessary—the idea being that it is ethically problematic for some (e.g., scientists) to be making decisions and taking actions that impose serious risks on others (i.e., members of the general public) without consent of, or adequate input from, the latter. Furthermore, because the consequences of GOFR are ultimately global in nature (i.e., GOFR conducted in one country can have risks and benefits for those living in other countries), many have emphasized the importance of greater international engagement, which is necessary to promote harmonization of GOFR governance. While it is widely accepted that expert scientific opinion is essential to well-informed GOFR decision- and policy-making, there have been calls for input from a wider range of scientific disciplines. Jonathan Suk and colleagues (2014), for example, argue that greater engagement with public health experts would facilitate both (1) assessment of GOFR risks and benefits and (2) design of GOFR studies that would have better translation into public health policy and practice. Many of these points are captured by the following statement of David Relman:

Woefully insufficient input has been obtained from a wide variety of scientists and from many other stakeholders among the general public. It is unethical to place so many members of the public at risk and then consult only scientists—or, even worse, just a small subset of scientists—and exclude others from the decision-making and oversight process … In many cases, conversations have only involved infectious-disease researchers and conflicts of interests among participants have not been adequately acknowledged or addressed … It is our responsibility as scientists to explain the rationale behind our work, including its benefits and risks, to the general public in terms that are accessible to those with an average level of education, rather than to be dismissive. This is especially important when the work has important consequences for the whole of society (Relman in Duprex et al. 2015, pp. 61–63).

There has likewise been broad support for the conduct—and transparent public dissemination—of GOFR risk-benefit analysis. Advocacy for risk-benefit analysis is partly motivated by recognition that any policy judgment that the benefits of any given GOFR study outweigh the risks (or vice-versa) should, insofar as possible, be evidence-based—and transparency is important because members of the public expect (and deserve) to be informed about the bases upon which key judgments/decisions are made (Fauci 2012).

While Kirsten Jacobson and colleagues suggest that, in light of measurement difficulties, “[a] qualitative risk-benefit analysis framework for assessing research…would be the most decisive tool for asking the hardest and most important questions” (Jacobson et al. 2014, p. 3), Marc Lipsitch and Thomas Inglesby argue that risk-benefit analysis can and should be quantitative because “[e]xtensive qualitative arguments have not provided sufficient clarity or evidence to resolve concerns or identify a consensus path forward … this process should be quantitative, rather than relying on unquantified and unverifiable assurances that particular laboratories are safe” (Lipsitch and Inglesby 2014, pp. 1, 5). Though they admit measurement challenges associated with objective quantitative risk-benefit analysis, Arturo Casadevall and Michael Imperiale (Casadevall and Imperiale 2014; Imperiale and Casadevall 2015) nevertheless argue that performing such analysis with the best available evidence could at least facilitate experimental designs that reduce risks or enhance benefits.

### Risk Measurement and Minimization

While it has long been acknowledged that biosecurity risks associated with dual-use life science research are especially difficult (if not impossible) to estimate with confidence (e.g., given unpredictable actions of potential malevolent actors) (Posner 2004), Lipsitch and Inglesby (2014) argue that the historical record of laboratory accidents at least enables evidence-based quantitative assessment of GOFR biosafety risks in particular. As summarized by Daniel Rozell (2015, p. 1), however, early attempts at quantitative GOFR risk assessment have lead to widely divergent estimations:

Using biosafety level 3 (BSL-3) lab infection data, Lipsitch and Inglesby [2014] estimated a probability of between 0.01 % and 0.1 % per laboratory-year of creating a pandemic which would cause between 2 million and 1.4 billion fatalities. This yields an expected fatality rate of 2000 to 1.4 million per BSL-3 laboratory-year. Alternatively, using data from the National Institutes of Allergy and Infectious Diseases, the probability of a pandemic would be between 0.05 % and 0.6 % per worker-year, with a resulting expected fatality rate of between 10,000 and 10 million per laboratory worker … A subsequent risk estimate from Fouchier [2015] started from the same data, but then Fouchier argued that [given special safety precautions taken in his H5N1 GOFR] a lab-induced pandemic would occur every 33 billion years—more than twice the known age of the universe.

Though further details of such calculations/analyses are beyond the scope of this paper, the risk-benefit assessment commissioned by the US Government will hopefully help resolve this controversy. In the meanwhile, even if Fouchier’s estimates about his own research are correct, which Lipsitch and Inglesby (2015) dispute, concerns about proliferation of GOFR conducted in less safe conditions should not be forgotten.

Despite this debate regarding the magnitude of biosafety risks posed by GOFR, there appears to be fairly widespread agreement that, other things being equal, research risks should be minimized (Casadevall et al. 2014a, b; Casadevall and Imperiale 2014; DHHS 2013; Duprex et al. 2015; Evans et al. 2015; Imperiale and Casadevall 2015; Lipsitch and Galvani 2014; Lipsitch and Inglesby 2014). It has been suggested that GOFR risks might be reduced via:

- Employment of safer pathogen strains
  - of low virulence,
  - for which there is immunity,
  - for which there are existing vaccines, and/or
  - which have been modified to inhibit replication outside of laboratories;
- Development/use of vaccines against experimental pathogen strains;
- Development/use of broad spectrum vaccines (e.g., pan- or universal influenza vaccine);
- Vaccination of laboratory workers to create a ring of immunity; and/or
- Ongoing improvement of biosafety practice and infrastructure.

Alternatively, it has been argued that research risks should be minimized via conduct of other less risky kinds of research rather than GOFR—at least in cases where the former would be equally beneficial in answering key scientific questions and/or achieving public health goals (see Lipsitch comments in Duprex et al. 2015; Evans et al. 2015; Lipsitch and Galvani 2014; Lipsitch and Inglesby 2014).

### Benefit Controversy

While the decision to publish the initial ferret H5N1 influenza studies of the research teams headed by Ron Fouchier and Yoshihiro Kawaoka (Herfst et al. 2012; Imai et al. 2012) in full was based on the judgment that benefits of publication outweighed the risks, numerous critics have questioned the actual benefits of these studies. Purported benefits of publication were that this would facilitate (1) development/production of vaccines against pandemic strains of the virus and (2) surveillance enabling early identification of, and thus response to, pandemic strains that might occur naturally. Critics have argued that such benefits are limited, *inter alia*, because naturally occurring pandemic strains may turn out be different from those created via the studies in question (in which case production of vaccines for, or surveillance targeting of, the latter might not be very useful); international surveillance systems are too weak “to detect a pandemic viral sequence … before it is too late” (Lipsitch and Galvani 2014, p. 3); “an important lesson learnt from pandemic H1N1 (swine flu) is that there is not much that can be done to contain outbreaks of pandemic strains of influenza once they emerge” (i.e., so early identification via surveillance might not make much difference) (Selgelid 2013, p. 148); and, given the way the vaccine industry actually works, there is unlikely to be development/stockpiling of vaccines against naturally-occurring transmissible strains of influenza before such strains actually arise (Selgelid 2013).

Lipsitch and Alison Galvani (2014) have additionally disputed the suggestion that these studies answered important public health questions—i.e., whether H5N1 might mutate into a human transmissible strain and what kinds of mutations might make this possible—in light of general difficulties translating ferret findings to humans (i.e., we cannot be sure that a strain of influenza transmissible in ferrets would be transmissible in humans) and complexities regarding epistasis (i.e., the phenotypic effects of any given mutation may depend on the broader genetic background of the organism in question; the same mutation may have different effects in different strains of a pathogen). In response to the point about translatability of ferret research to humans, Imperiale and Casadevall (2015) have responded that if this is a reason to be skeptical about benefits then it is also a reason to be skeptical about risks associated with the research in question.

While the reality or magnitude of risks associated with dual-use and GOF research have frequently been questioned (e.g., is malevolent use a tangible/significant threat or merely a theoretical possibility?), Nicholas Evans has argued that purported benefits of dual-use and GOF research should likewise not be simply taken for granted. Whether or not theoretically possible benefits of any given study are realized, according to Evans, will depend on background institutional factors (e.g., strength of healthcare infrastructure(s), systems of surveillance and countermeasure production, political will and resources necessary to translate scientific findings into benefits) that may or may not exist and/or may vary widely from country to country (Evans 2013, 2014a, b; Evans et al. 2015).

This last point highlights justice implications of GOFR—i.e., because some (people or countries) will be better able to protect against risks and/or realize benefits from GOFR than others. Alta Charo, for example, argues that:

the benefits [of GOFR] will disproportionately go to people who are either personally better off or in wealthier countries because that is often where the healthcare system or economic access to healthcare is better. We need to pay more attention to making sure that the benefits are justly distributed and the science is beneficial for everybody (NRC and IOM 2015, p. 66).

Casadevall and colleagues (2014a, b) emphasize potential epistemic benefits of GOFR. They argue that the controversial H5N1 ferret research, employing well-established scientific methodology, provided the only way to demonstrate with certainty the possibility that H5N1 “had the biological capacity to generate variants that could spread from mammal to mammal” (2014a, p. 2). Acknowledging that potential benefits of advances in scientific knowledge may be long term—and difficult to predict ahead of time—they nonetheless maintain that GOFR benefits in the way of knowledge production should be taken into consideration, and not underestimated, in risk-benefit analysis of GOFR. Evans (2014a) concurs that scientific knowledge is valuable, but argues that appropriately factoring scientific knowledge advancement into risk-benefit analysis requires clarity regarding whether or not, or the extent to which, knowledge should be considered intrinsically valuable (i.e., valuable for its own sake) as opposed to merely instrumentally valuable (i.e., valuable only insofar as it promotes realization of other things of intrinsic value).

Given the value of scientific knowledge advancement, numerous authors have warned about various ways in which GOFR controversy could stall important areas of scientific development (Casadevall and Imperiale 2014; Duprex et al. 2015; Evans et al. 2015; Fauci 2012; Imperiale and Casadevall 2015; Lipsitch and Inglesby 2014; Pfeiffer 2015; Suk et al. 2014; Wain-Hobson 2014). An untoward event could lead to societal backlash, for example, and/or increased regulations may discourage scientists from pursuing certain kinds of research. Such worries highlight one reason, among many, why good governance of GOFR is crucial.

Arguing that (1) numerous other kinds of scientific research and/or public health activities would be equally (or more) beneficial in answering key scientific questions and/or promoting public health goals and (2) GOFR creation of potential pandemic pathogens (PPPs) poses large risks to large numbers of people, Lipsitch and co-authors conclude that (3) the benefits of GOFR creation of PPPs do not outweigh the risks, and thus that GOFR creation of PPPs should be considered unjustified (unless, at least, objective quantitative risk-benefit analysis proves otherwise). In reaching this conclusion, they appeal to Nuremburg Code and Belmont Report requirements that research should “be done only if it benefits society, if the same benefits could not be procured through less risky means, and if the anticipated benefits exceed the anticipated risk” (Evans et al. 2015). Though they acknowledge that the Nuremburg Code and Belmont Report were explicitly designed to govern research involving human subjects, they argue that (in light of the general ethical considerations upon which such guidelines are based) these requirements have broader applicability to risky research more generally. While Lipsitch and co-authors advocate quantitative GOFR risk-benefit analysis, they emphasize the importance of assessing GOFR studies “on the basis of their marginal benefits, compared to those of safer approaches” (the idea being that any increased risks must be outweighed by increased benefits in order for GOFR studies to be justified) (Lipsitch and Galvani 2014, p. 5).

## Risk-Benefit Assessment of Gain-of-Function Research

As part of the deliberative process called for during the pause on selected gain-of-function research involving influenza, MERS, and SARS viruses, the US Government has commissioned an in-depth, systematic assessment of the risks and benefits specifically associated with this kind of research. In its *Framework for Conducting Risk and Benefit of Gain*-*of*-*Function Research*, NSABB (2015) has recommended that the contractor responsible for this work assess the following kinds of potential (possibly overlapping) risks and benefits, including security implications thereof, in particular:

### Risks

- Biosafety—i.e. dangers associated with laboratory accidents;
- Biosecurity—i.e., dangers associated with crime and terrorism if pathogens are not physically secure and/or if malevolent actors gain access to them;
- Proliferation—i.e., dangers that might grow proportionally with an increased rate of GOFR, potentially in different settings with varying biosafety standards;
- Information risk—i.e., if published studies facilitate malevolent action (e.g., by terrorists) or, possibly, breach of intellectual property;
- Agricultural—i.e., risks to agriculturally-relevant animals if enhanced pathogens arising from GOFR are accidentally or intentionally released into animal populations, and possible implications for human health;
- Economic risks—i.e., financial implications of (accidental or intentional) pathogen release or, possibly, opportunity costs; and
- Loss of public confidence—i.e., compromise of trust in the scientific enterprise that could result from (accidental or intentional) pathogen release.

### Benefits

- Scientific knowledge—i.e., (potentially unique) information gained, and the value of such information for understanding pathogens/disease;
- Biosurveillance—i.e., enhancement of (a) public health surveillance, (b) agricultural and domestic animal surveillance, and (c) wildlife surveillance—to improve outbreak detection/prediction and/or decision-making;
- Medical countermeasures—i.e., (potentially unique) information facilitating development of therapeutics, vaccines, and diagnostics;
- Informing policy decisions—i.e., regarding public health preparedness (e.g., countermeasure stockpiling, vaccine strain selection, resource mobilization); and
- Economic benefits—i.e., financial gains (e.g., from industrial productivity) and/or cost savings (e.g., from reduced health care expense).

The conduct and dissemination of findings from this risk-benefit assessment (RBA) will (1) address a demand expressed by commentators in debate surrounding GOFR (i.e., that RBA is conducted and made public), (2) hopefully help resolve controversy surrounding the extent of risks and/or benefits of GOFR (e.g., empirical debates about the magnitude of biosafety risks discussed in the above literature review), and (3) inform policy-making regarding the funding and conduct of GOFR.

### Risk-Benefit Assessment Limitations

While the commissioned RBA will be valuable in all of these ways, it may be a mistake to think that RBA will provide a panacea for solving difficult policy issues surrounding GOFR. Though RBA could undoubtedly promote better informed policy decisions (and thus better policy decisions), for example, it is perhaps unlikely that RBA will itself provide a clear guide to action regarding the funding and conduct of GOFR.^2^ This is for numerous reasons.

#### Complexity and Uncertainty

First, given the inordinate complexities involved with assessing the risks and benefits of GOFR—considering, for example, all the possible scenarios for better or worse that might arise, and the enormous number of factors that outcomes depend on—it would be difficult for RBA to reveal, with a high degree of confidence anyway, the likelihood and magnitude of harms and/or benefits that could result from GOFR. A widely acknowledged limitation of RBA is that confidence in predictions generated depends upon the quality of (1) input data and (2) models employed in assessment of risks and benefits.^3^ Both data and models will inevitably be imperfect in the context of GOFR in light of scenario complexity, uncertainties, unknown unknowns, and presumably *unknowable* unknowns, that are relevant to GOFR consequences. The likelihood and magnitude of harms that could result from GOFR, for example, partly depend upon the actions of malevolent actors. There are innumerable possible actions that such actors might take, however—and the likelihood of any given action and/or the consequences thereof (given all the relevant factors involved) are arguably inestimable (Posner 2004). In some cases the commissioned RBA will aim to provide qualitative rather than quantitative analysis—precisely because the latter will not always be feasible. At the September 2015 meeting of the NSABB, Rocco Casagrande (Managing Director of Gryphon Scientific, which has been commissioned to complete the RBA currently underway) explained that assessment of potential benefits of GOFR (e.g., regarding countermeasure development) will be qualitative rather than quantitative because there is inadequate data for the latter (Casagrande 2015).^4^

RBA will, despite challenges noted above, hopefully provide the best assessments possible, acknowledging limitations regarding both quantitative and qualitative findings—and this would provide valuable input to decision-making processes. It is better to make informed rather than uninformed policy decisions regarding GOFR—and we can only inform ourselves to the best of our ability. To the degree that findings are uncertain (because based on imperfect data, estimates, and/or models), however, they may need to be considered with caution.

#### When Do Benefits Outweigh Risks and Vice Versa?

Quantification

Second, the findings of RBA might not themselves reveal whether expected benefits actually outweigh expected risks, or vice versa. This is partly because, as noted above, not all expected risks and benefits will be quantified by the RBA endeavor currently underway. Unless potential benefits of GOFR are quantified (e.g., in terms of the expected number of lives saved—given the likelihood and extent of life-saving that may result from potential improvement of countermeasures), it may not be obvious whether they outweigh quantified risks (e.g., in terms of expected number of lives lost—given the likelihood and severity of possible untoward outcomes resulting from GOFR).^5^

Values and Weightings

Even if all assessed risks and benefits were in fact quantified with a high degree of confidence, this may still not determine whether benefits outweigh risks, or vice versa, because that would depend on how benefits (or the ultimate values they promote) should be weighed against risks (or the ultimate values they compromise). *Inter alia*, this reveals the need for distinguishing things that are merely instrumentally valuable (i.e., valuable because they promote what is intrinsically valuable) from things that are intrinsically valuable (i.e., valuable for their own sake). Whether or not benefits outweigh risks, or vice versa, ultimately depends on whether there is (expected to be) net gain or loss of that which is intrinsically (or ultimately) valuable. To itself provide a clear guide to action, RBA would thus need to quantify or otherwise assess ultimate implications of GOFR regarding that which is intrinsically (or ultimately) valuable.

Many of the benefits and risks to be evaluated by RBA are presumably merely instrumentally valuable. Medical countermeasures, surveillance, and economic gains, for example, are arguably largely valuable not for their own sakes but in virtue of the role they play in protecting and/or promoting human well being (in the way of public health).^6^ Presumably almost everyone will agree that human well being (in the way of public health) is one of the things that ultimately matters for its own sake, and thus one of the things that policy should ultimately aim to promote.^7^

The nature of other values associated with potential risks and benefits of GOFR, on the other hand, might not be so clear. There may be reasonable disagreement, for example, about whether the gain of scientific knowledge is merely instrumentally valuable, or also valuable for its own sake (Kitcher 2001; Evans 2014a). Similar things might be said about the value of security, which looms large in debates about GOF research. Policy debates about dual use research more generally have often been framed in terms of potential conflict, and/or the need to strike a balance, between the value of security, on the one hand, and the value of scientific progress, and the good things thereby enabled, on the other. In its *Framework for Conducting Risk and Benefit of Gain*-*of*-*Function Research* NSABB (2015) has recommended that the RBA contractor consider the security implications of the kinds of risks and benefits enumerated above. Conceived as the “protection of valuable things against loss” (Selgelid 2012), security can be considered a meta-value. Protection of valuable things against loss can include both protection of instrumentally valuable things against loss and protection of intrinsically valuable things against loss. In the latter case, the value of security pertains to the good of society writ large. Among other things, the ultimate good of society arguably consists in (aggregate) human well being, liberty, equality, and our democratic way of life. All of these values could potentially be compromised by pandemic risks that GOFR might reduce or exacerbate. While security (conceived as protection of such things against loss) is thus especially important, there might be reasonable disagreement about whether or not, or the extent to which, security is intrinsically valuable, or merely valuable insofar as it plays a role in promoting such things. This is an important (rather than merely academic) matter because it raises the question of whether or not, or the extent to which, it would be legitimate to make net sacrifices of (other things of) intrinsic value in order to gain more security.^8^

Part of the purpose of the discussion above is to reveal complexity surrounding the anatomy of values, and the importance of clarity regarding value hierarchy. Determining whether benefits of GOFR outweigh risks requires (1) distinguishing things that are intrinsically valuable from those that are merely instrumentally valuable and (then) (2) determining whether GOFR (or any particular case of GOFR) would lead to net benefit regarding the former kinds of goods in particular. RBA, however, will not settle questions about which goods pertaining to risks and benefits of GOFR are intrinsically valuable, because this is a matter of ethics rather than empirical science.

Even if a list of intrinsically valuable goods were taken as given, additional difficult ethical questions arise. First is the question of how potentially conflicting intrinsic goods should be weighed against one another—e.g., if GOFR would promote net gains in terms of some (e.g., aggregate well being) at net cost in terms of others (e.g., individual liberty in the way of freedom from significant risks in the absence of consent). Second is the question of the weight that should be given to benefits that may arise in the future—i.e., what, if anything, should the “future discount rate” be in the event that GOFR entails significant risks at present in order to achieve net benefits in the future (and/or for future generations) (Murray 1994). Third, and especially important, is the question of risk aversion, risk appetite, and/or risk-taking strategy. It is common to place greater disvalue on losses than value on gains (e.g., in things like well being or money) of equal magnitude, and it is not obviously irrational to do so. Whether or not benefits of GOFR are thought to outweigh risks may thus (depending on RBA findings) partly depend on what is considered appropriate risk-taking strategy (e.g., to what extent, if any, should decision-making reflect risk aversion?). Different risk-taking strategies embodying different levels of risk aversion may yield different answers to questions about what should be done if RBA reveals that GOFR (or a certain case thereof) is reasonably likely to promote a significant amount of human well being (e.g., by facilitating disease control) but has a very small chance of leading to catastrophic consequences (e.g., in the event of laboratory accident or malevolent use of research findings).

## Existing Ethical and Decision-Making Frameworks

The above-mentioned limitations of RBA highlight the importance of ethical input to decision- and policy-making regarding the funding and conduct of GOFR. Such decision- and policy-making ultimately concerns questions about what should (or ought to) be done in light of information provided by RBA; and questions about what should (or ought to) be done is, by definition, what the discipline of ethics aims to address. This section outlines a variety of existing ethical and decision-making frameworks that might be brought to bear on decision- and policy-making regarding GOFR.

### Decision Theory

#### Expected Utility Maximization

A well-developed, and much discussed, approach to decision-making in contexts of risk holds that it would be rational to choose the action (or policy) with *maximum expected utility*, where the expected utility of any given action (or policy) is defined as the sum of the products of the likelihood and utility (or value) of each possible outcome of that action (or policy). Suppose, for example, that there are two options with the following possible consequences:

- Option A, which has two possible outcomes:
  - There is a 50 % (or .5) chance that Option A will lead to outcome A1, which embodies UA1 amount of utility (or value).
  - There is a 50 % (or .5) chance that Option A will lead to outcome A2, which embodies UA2 amount of utility (or value).
- Option B, which has 3 possible outcomes:
  - There is a 60 % (or .6) chance that Option B will lead to outcome B1, which embodies UB1 amount of utility (or value).
  - There is a 30 % (or .3) chance that Option B will lead to outcome B2, which embodies UB2 amount of utility (or value).
  - There is a 10 % (or .1) chance that Option B will lead to outcome B3, which embodies UB3 amount of utility (or value).

The expected utility of Option A (EUA) and the expected utility of Option B (EUB) would be calculated as follows:

$$ \begin{aligned} {\text{EUA}} & = \left( {.5 \times {\text{UA}}1} \right) + \left( {.5 \times {\text{UA}}2} \right) \\ {\text{EUB}} & = \left( {.6 \times {\text{UB}}1} \right) + \left( {.3 \times {\text{UB}}2} \right) + \left( {.1 \times {\text{UB}}3} \right) \\ \end{aligned} $$

According to the expected utility maximization approach to decision-making, it would be rational to choose Option A if EUA is greater than EUB; and it would be rational to choose Option B if EUB is greater than EUA.

Suppose, hypothetically, that RBA findings regarding risks and benefits of (a particular case of) GOFR involving H5N1 avian influenza virus reveal that we are ultimately faced with the following choice situation^9^:

- Option 1: Refrain from GOFR, which would entail the following possible outcomes:
  - There is a 10 % (.1) chance that H5N1 naturally mutates into a pandemic strain that kills 100,000,000 people (in the absence of improved control measures that might have been possible via GOFR).
  - There is a 90 % (.9) chance that no H5N1 pandemic occurs, so no lives are lost.
- Option 2: Pursue GOFR, which would entail the following possible outcomes:
  - There is a 5 % (.05) chance that H5N1 naturally mutates into a pandemic strain that kills 100,000,000 people (because GOFR does not lead to improved control measures).
  - There is a 5 % (.05) chance that H5N1 naturally mutates into a pandemic strain that kills only 40,000,000 people (because GOFR results in effective new control measures).
  - There is a .6 % (.006) chance that laboratory accident or malevolent action leads to an H5N1 pandemic (involving a strain that might have occurred naturally) killing 100,000,000 people (because GOFR has not, or not yet, lead to effective new control measures).
  - There is a .4 % (.004) chance that laboratory accident or malevolent action leads to an H5N1 pandemic (involving a strain that might have occurred naturally) killing only 40,000,000 people (because GOFR results in effective new control measures).
  - There is a .06 % (.0006) chance that laboratory accident or malevolent action leads to an H5N1 pandemic (involving a strain more dangerous than would have arisen naturally) killing 2,500,000,000 people (because GOFR has not, or not yet, lead to effective new control measures).
  - There is a .04 % (.0004) chance that laboratory accident or malevolent action leads to an H5N1 pandemic (involving a strain more dangerous than would have arisen naturally) killing (only!) 1,000,000,000 people (because GOFR results in effective new control measures).
  - There is an 88.9 % (.889) chance that no H5N1 pandemic occurs, so no lives are lost.

Assuming that utility/value is determined by number of lives lost, then the expected utility of Option 1 (i.e., refraining from GOFR) would be:

$$ \left( {.1 \times 100{,}000{,}000\;{\text{lives}}\;{\text{lost}}} \right) + \left( {.9 \times 0\;{\text{lives}}\;{\text{lost}}} \right) = 10{,}000{,}000\;{\text{lives}}\;{\text{lost}} $$

The expected utility of Option 2 (i.e., pursuing GOFR) would be:

$$ \begin{aligned} & \left( {.05 \times 100{,}000{,}000\;{\text{lives}}\;{\text{lost}}} \right) + \left( {.05 \times 40{,}000{,}000\;{\text{lives}}\;{\text{lost}}} \right) + \left( {.006 \times 100{,}000{,}000\;{\text{lives}}\;{\text{lost}}} \right) \\ & + \left( {.004 \times 40{,}000{,}000\;{\text{lives}}\;{\text{lost}}} \right) + \left( {.0006 \times 2{,}500{,}000{,}000\;{\text{lives}}\;{\text{lost}}} \right) \\ & + \left( {.0004 \times 1{,}000{,}000{,}000\;{\text{lives}}\;{\text{lost}}} \right) + \left( {.889 \times 0\;{\text{lives}}\;{\text{lost}}} \right) = 9{,}660{,}000\;{\text{lives}}\;{\text{lost}} \\ \end{aligned} $$

According to the expected utility maximization approach to decision-making, we should thus choose Option 2—i.e., proceed with GOFR—because this would lead to a smaller number of expected lives lost.

There are some kinds of cases where an expected utility approach to decision-making might obviously be rational and prudent. Suppose one was offered the following gamble: A fair die is tossed and you receive $7 if it lands on number 6, and you pay $1 if it lands on any other number. The expected utility of not taking this gamble would be $0—i.e., you would not gain or lose any money.^10^ The expected utility of taking this gamble would be:

$$ \left( {5 /6 \times - \$ 1} \right) + \left( {1 /6 \times \$ 7} \right) = \$ 0.33 $$

According to the expected utility approach to decision-making, one should take the gamble. Assuming that one is not morally opposed to gambling, and that one could play the game as often as one likes, furthermore, it would presumably be rational to do so—because one could expect to win an average of $0.33 per roll of the die.

According to the expected utility maximization approach to decision-making, however, one should take a gamble like this even if it were only offered once—because the expected utility of playing would still be greater than the expected utility of not playing. If one could only play a game like this once, however, then it is highly likely (i.e., there is a 5 in 6 chance) that one would end up losing—so it is not so obvious that it would be irrational or imprudent to refrain from playing. Assuming one can afford to lose $1, on the other hand, it would likewise not obviously be irrational to take one shot at a game like this.

Another, related kind of challenge to the expected utility maximization approach to decision-making (and one that might be especially relevant to GOFR) is revealed by imagining a similar kind of gamble with higher stakes: A fair die is tossed and you pay $100,000 if it lands on number 6, and you win $20,001 if it lands on any other number. The expected utility of taking this gamble would be:

$$ \left( {1 /6 \times - \$ 100,000} \right) + \left( {5 /6 \times \$ 20,001} \right) = \$ 0.83 $$

Despite the positive expected utility of such a gamble, taking it would be considered (highly) irrational by almost everyone (or at least those without millions of dollars to gamble with). For many people, such a gamble would ultimately involve betting one’s house, with a fairly high (i.e., 1 in 6) chance of losing it. This objection to the expected utility maximization approach to decision-making is that it might sometimes be rational/prudent to sacrifice expected utility in order to avoid options with especially costly possible outcomes. The underlying suggestion is that the expected utility maximization approach to decision-making is not sufficiently risk averse. The aim to avoid options with especially costly possible outcomes (even when the option in question maximizes expected utility) gives rise to doubt that GOFR should actually be pursued in the hypothetical example above—the idea being that it would be too risky to pursue a course of action that has a nontrivial possibility of killing 2,500,000,000 people even if expected utility would be maximized by such a course of action.^11^

In any case, it would presumably be impractical to employ the expected utility maximization approach to decision-making in the context of GOFR policy-making—because such an approach requires (1) identification of all the possible outcomes of options, (2) estimation of the likelihood of such outcomes, and (3) estimation of the utility (or value) of each outcome. For reasons discussed above, this would be unrealistic in the case of GOFR (see also Douglas 2013; Resnik 2014). It is impossible to predict, with any confidence, the likelihood of malevolent use (Posner 2004), for example, and there are innumerable scenarios that could result from such use.

#### Maximin

Another approach to decision-making involves the idea that we should identify the worst possible outcome that might arise from each option under consideration and then choose the option with the best worst possible outcome—i.e., we should choose the option for which the worst outcome is least bad, or we should aim to maximize the utility of the possible outcome with the minimum utility. It is commonly thought that such an approach, referred to as the maximin risk-taking strategy, would be especially appropriate in circumstances where the probability of outcomes that might arise from various options is unknown, but a risk-taking strategy like this could also be considered an alternative to the expected utility maximization approach to decision-making even in cases where the probabilities of option outcomes are estimable. In the latter kind of case, for example, the maximin strategy would call for a decision to refrain from GOFR in the hypothetical H5N1 example considered above, because the worst possible outcome of GOFR (2,500,000,000 lives lost) is worse than the worst possible outcome of refraining from GOFR (100,000,000 lives lost). The maximin strategy also captures the intuition that it would be irrational (for those who are not millionaires anyway) to take the high stakes die gamble.

While the maximin strategy addresses the concern that the expected utility maximization approach to decision-making is insufficiently risk averse, the maximin strategy arguably goes too far in the opposite direction (Hansson 2003). The hypothetical example regarding H5N1 considered above, for example, was designed to suggest that pursuing (at least certain kinds of) GOFR should be considered the option with the worst possible outcome, because (certain kinds of) GOFR might entail the possibility of disaster resulting from pathogens more dangerous than those that otherwise would have arisen. Even when/if this is correct, however, it is not obvious that this should imply that (such cases of) GOFR should never be pursued. Even if we assume that the worst possible outcome of (a certain case of) GOFR is worse than the worst possible outcome of refraining from GOFR, we might nonetheless think that GOFR should be pursued. One could imagine a case of GOFR that:

- is highly likely to have enormous benefits;
- has a worst possible outcome considered to be extremely unlikely (though likelihood of the worst possible outcome may be uncertain and/or exceedingly difficult to estimate with confidence);
- has a worst possible outcome that is not considered to be more likely—and/or is considered to be less likely—than the worst possible outcome of refraining from GOFR (though likelihood of the worst possible outcome of refraining from GOFR is likewise uncertain and/or exceedingly difficult to estimate with confidence);
- has a worst possible outcome that is only just slightly worse than the worst possible outcome of refraining from GOFR.

Though a maximin approach would call for refraining from GOFR in such a case, it is by no means clear that this would be appropriate. A problem with the maximin approach is that it requires maximization of the utility of the worst possible outcome regardless of (1) the cost in terms of forgone benefits, (2) the likelihood (uncertain or otherwise) of the worst possible outcomes of alternative actions, and (3) the extent to which the worst outcome of the option with the best worst outcome is actually better than the worst outcomes of other options.

#### Maximax

The maximax approach is the polar opposite of maximin. It holds that we should choose the option with the best possible outcome—i.e., we should choose the option for which the best possible outcome embodies the greatest amount of utility, or we should aim to maximize the utility of the possible outcome with maximum utility. Though less widely discussed than the approaches presented above, there are cases where such a decision-making strategy might be considered preferable to either maximin or the expected utility maximization approach to decision-making. One might imagine a case of GOFR that:

- has an expected utility that is slightly less than the expected utility of refraining from GOFR;
- has a worst possible outcome that is slightly worse (and not significantly more likely) than the worst possible outcome of refraining from GOFR;
- has a possible outcome that is much better than any possible outcome of refraining from GOFR (e.g., it is highly unlikely, but possible, that the GOFR in question will lead to a broad spectrum influenza vaccine that prevents enormous numbers of deaths for years to come).

Proceeding with GOFR in a case like this—i.e., following a maximax strategy—might not obviously be inappropriate. Maximax is an ambitious risk-taking strategy that embodies the idea “nothing ventured, nothing gained” (Sunstein 2005). It is arguably the strategy behind at least some blue-sky research—and the (not obviously irrational) strategy employed by those who play lotteries—which usually involve negative expected utility and the worst bad outcome (i.e., loss of a dollar or two) but provide the chance of winning a not otherwise attainable fortune. On the other hand, it is also easy to imagine cases where such an approach would obviously be irrational/imprudent.^12^

#### Pluralism

Maximum expected utility, maximin, and maximax, might each be legitimate goals. Other things being equal, that is, decision-making should arguably favor the option with maximum expected utility. Other things being equal, decision-making should arguably favor the option with the best worst outcome (maximin). And, other things being equal, decision-making should arguably favor the option with the best possible outcome (maximax).

There may be cases where the very same option promotes all three of these things (maximum expected utility, maximin, and maximax) at the very same time—and in cases like that (which could turn out to include cases of GOFR) it might be quite obvious what should be done. In other cases there might be conflict between these three arguably legitimate goals of decision-making. Such cases raise difficult questions about the weightings that should be attributed to such goals and/or how to strike a balance, or make trade-offs, between them. The hypothetical examples discussed above suggest that the weightings attributable to such goals may be context dependent—e.g., maximin might be especially weighty in high risk situations, maximax might be especially weighty in low risk situations, and expected utility maximization might be especially weighty in cases were multiple attempts (at the gamble in question) are possible and/or in low stake situations (i.e., where the worst possible outcome is not so bad). Different risk-taking strategies (employed by different people), in any case, might attach different weightings to the goals in question—and there may be reasonable disagreement about what, if any, is the correct risk-taking strategy. In a democracy, the risk-taking strategy employed by policy-making should arguably reflect the risk-taking strategies of the people.

### Precautionary Approach

The “precautionary principle”, or versions thereof, has often been appealed to in contexts of uncertainty and catastrophic risk, and debates about environmental dangers in particular. A relatively weak, and not especially controversial, version of the precautionary principle is adopted by the *Rio Declaration on Environment and Development*:

Where there are threats of serious or irreversible damage, lack of full scientific certainty shall not be used as a reason for postponing cost-effective measures to prevent environmental degradation (United Nations Conference on Environment and Development 1992).

This version of the precautionary principle is partly a claim about burden of proof, the idea being that we need not have certainty that a given course of events will lead to great harm in order to justify taking preventative action against the potential dangers in question. In the context of GOFR, such a version of the precautionary principle would entail that uncertainty about dangers regarding biosafety and/or malevolent use would not provide reason (e.g., in decision- and policy-making) to ignore the potential dangers in question. This version of the precautionary principle is considered relatively weak, however, because it does not clearly imply an especially high degree of risk aversion, and it would not (necessarily) rule out potentially risky GOFR.

Stronger versions of the principle, however, are (akin to the maximin approach) more clearly risk averse. The strongest version of the precautionary principle would hold that we should not take actions that pose serious dangers (where likelihood of the dangers in question is uncertain). Cass Sunstein (2005) argues that such a strong version of the principle would be incoherent, because serious dangers will be possible outcomes of any course of action. In the context of the environment, it might be thought that this strongest version of the precautionary principle speaks against developing and/or releasing genetically modified organisms (GMOs), because their development/release might pose serious (though, admittedly, uncertain) dangers. Sunstein (2005), however, has noted that the failure to develop and/or release GMOs might likewise pose serious (though, admittedly, uncertain) dangers, because it might turn out that GMOs enable avoidance of major famines that would otherwise occur. The strongest version of the precautionary principle would thus apparently (also) entail that we should not refrain from GMO development/release. In the context of GOFR one might argue that, according to the strongest version of the precautionary principle, we should not pursue GOFR because GOFR may lead to serious dangers involving laboratory accidents or malevolent use. By the same token, however, one might argue that we should pursue GOFR because GOFR might enable control of pandemics that would otherwise be disastrous. The strongest version of the precautionary principle thus appears to give conflicting advice regarding both GMOs and GOFR, and thus no guidance at all. Sunstein argues that people’s appeal to the strongest version of the precautionary principle can be explained by the fact that they are more attuned to some kinds of dangers than others, due to cognitive bias.^13^

More moderate versions of the precautionary principle hold that we should avoid actions (or courses of action) when the dangers they pose are not merely serious, but exceed severity thresholds. Sunstein (2005), for example, appeals to an Anti-Catastrophe version of the precautionary principle according to which we should avoid courses of action that pose catastrophic dangers in particular. Because catastrophe might possibly result from any course of action he furthermore argues that the likelihood of catastrophe, though uncertain, would need to exceed a likelihood threshold—i.e., the catastrophic danger in question, though uncertain, would need to be sufficiently likely (as opposed to a theoretical or minutely remote possibility)—in order for the Anti-Catastrophe version of the principle to take effect. Though moderate versions of the precautionary principle like this might be more plausible than strong versions of the precautionary principle, questions remain regarding how likely a catastrophic risk would need to be in order for such a principle to take effect (i.e., where, exactly, should the likelihood threshold be set?). It also raises questions about the magnitude of harm that should divide catastrophic (or, in other moderate versions of the precautionary principle, sufficiently serious) dangers from others. Different risk-taking strategies, embodying different levels of risk aversion, will set such thresholds in different places.

Even moderate versions of the precautionary principle might arguably, depending on where thresholds are set, be implausibly risk averse—i.e., by entailing that there are certain courses of action that we should never pursue regardless of their expected benefits. Moderate versions of the precautionary principle, finally, like stronger versions, might sometimes provide conflicting guidance (and thus be incoherent/paradoxical) (Clarke 2013). If both (a certain case of) GOFR and the failure to pursue (a certain case of) GOFR pose nontrivial though uncertain dangers of catastrophe beyond thresholds for likelihood and severity of harm, then even moderate versions of the precautionary principle, such as that advocated by Sunstein, would entail both that we pursue and that we refrain from pursuing the GOFR in question.

Frida Kuhlau and colleagues (2011) have developed/proposed a specific version of the precautionary principle, for dual-use life science research in particular, that holds:

When and where serious and credible concern exists that legitimately intended biological material, technology or knowledge in the life sciences pose threats of harm to human health and security, the scientific community is obliged to develop, implement and adhere to precautious measures to meet the concern (p. 8).

While David Resnik (2013) likewise appeals to the precautionary principle in the context of dual-use research, on his view

the basic idea of the precautionary principle is that we should take *reasonable* measures to avoid, minimize, or mitigate harms that are plausible and serious (p. 28, my emphasis).

In responses to Kuhlau and colleagues, that might also apply to Resnik, Steve Clarke highlights the importance of clarity regarding the role that the precautionary principle is meant to play in decision- and policy-making. Precautionary principles described by Kuhlau and colleagues and Resnik might sound reasonable if they are meant to supplement rather than replace cost-benefit analysis (CBA)^14^; but why, asks Clarke (2013, pp. 231–232), should we think that CBA requires such supplementation to begin with? Would a cost-benefit approach to dual-use life science research (and/or GOFR) deny that such research poses plausible serious risks warranting serious remedies and/or exclude such risks from consideration; and is appeal to the precautionary principle thus necessary to address an actual gap in CBA?

If Kuhlau and colleagues intend to suggest a stronger precautionary principle that is meant to replace (rather than merely supplement) CBA, on the other hand, then Clarke argues that their principle would (like other strong versions of the precautionary principle discussed above) be (1) implausibly insensitive to forgone benefits associated with precautionary action and (2) likely to give conflicting guidance.

### Rights-Based Approach

Beyond utility risks and benefits, a crucial point of Sven Ove Hansson (2003) is that equity and rights are essential to risk-related decision- and policy-making. Just as it would (usually) be rights-violating and thus unethical for one person (or group) to harm another, it might at first glance be thought that it would be unethical for one person (or group) to impose risk of harm on others (in the absence of explicit consent). Because ordinary action—e.g., driving one’s car down the street (without explicit consent of residents)—involves imposing risks on others, however, it cannot be the case that every instance of risk imposition on others (in the absence of explicit consent) constitutes unethical action (Hansson 2003). We mutually benefit by allowing one another to impose (certain) risks on each other (in the absence of explicit consent); and if imposition of risks on others were ruled out, by ethics or policy, then human life would come to a standstill. This raises the question of what should be considered ethically acceptable risk imposition. Hansson argues that

[while] everyone has a prima facie moral right not to be exposed to risk … this right can be overridden if [and only if] the risk-exposure is part of an equitable system for risk-taking that works to the advantage of the individual risk-exposed person (p. 291).

This kind of approach is preferable to the underlying utilitarian thinking behind expected utility maximization, according to Hansson, because the latter kind of approach (in addition to other objections raised above) is insensitive to human rights and distributive/egalitarian concerns.^15^ Risks associated with GOFR, according to Hansson’s approach, might be considered acceptable if the scientific and technological enterprise (if that is meant to be the “system of risk-taking” in question) equitably benefited all of those exposed to the risks involved. Given global political economics, some might doubt that this is the case—because some people exposed to the risks involved benefit more from scientific and technological advance than others. If Hansson’s principle is taken to be absolute, then risks associated with the scientific and technological enterprise would be considered unacceptable if such doubts about equity are justified. If a degree of inequity is inevitable (in light of global political economics) but the scientific and technological enterprise nonetheless perhaps enormously benefits the vast majority of (though not all) people exposed to the risks involved, then one might think that the risk imposition in question is actually justified. If Hansson’s principle is absolute, then it would apparently always prioritize equity over utility, but it is plausible that small compromises regarding equity are at least sometimes outweighed by large utility gains (Selgelid 2009b). A more moderate (and less binary) principle than that defended by Hansson (but which nonetheless remains sensitive to rights and distributive/egalitarian concerns) might run as follows: while everyone has a prima facie moral right not to be exposed to risk, over-riding of this right is ethically acceptable *to the degree that* risk-exposure is part of an equitable system for risk-taking that works to the advantage of risk-exposed persons. Such a principle would be more tolerant of risks associated with the scientific and technological enterprise (and thus at least some cases of GOFR).

### Deontological Ethics and Double Effect

Deontological approaches to ethics hold that some actions—e.g., intentionally killing an innocent person—would never be morally permissible regardless of the consequences of the action in question. Given the relevance of intentions to the moral permissibility of actions according to (many) deontological ethical frameworks, the “doctrine of double effect” (DDE) is meant to provide “a guide to decision making in ethically difficult cases where an action or course of action with an intended good effect can also produce a foreseen bad effect” (Uniacke 2013, p. 153). DDE holds that it may be morally permissible to pursue an action with a foreseen bad effect so long as the action in question is not itself morally problematic, the bad effect is not itself intended (it is merely foreseen), an intended good effect is directly produced by the action in question (and not directly produced by the bad effect), and this intended good effect outweighs the foreseen bad effect (the proportionality condition) (Uniacke 2013, p. 155). Suzanne Uniacke illustrates the application of DDE to a case where a driver must swerve a car into the path of an innocent pedestrian in order to avoid crashing into a crowd. According to DDE, this might be ethically permissible because the killing of the pedestrian is merely foreseen rather than intended, the saving of the crowd is brought about by the swerving of the car (rather than being caused by the death of the pedestrian), and the many lives saved outweigh the one life lost.

As demonstrated by Uniacke, there are obvious similarities between scenarios where DDE is commonly invoked and the dual-use problematic.^16^ In the context of dual-use research, responsible scientists (and/or their funders) intend to conduct (or enable) work that will be benefit humanity (i.e., produce good effects); but they may foresee, though they do no intend, that malevolent use of the research may lead to grave harm (i.e., produce bad effects). Should DDE thus apply to dual use dilemmas? This partly depends on whether DDE is a plausible principle—which has been the subject of much ethical controversy.^17^ In any case, Uniacke points out numerous differences between scenarios where DDE is commonly thought to apply and dual use dilemmas:

- In prototypical DDE scenarios the foreseen bad effect is (usually) expected with certainty or high probability, but in the dual use context bad effects are merely a foreseen possibility (and/or presumably often considered to be low probability).
- In prototypical DDE scenarios the foreseen bad effect is (usually) directly produced by the moral agent in question, but in the dual use context the foreseen possible bad effect would result from the malevolent action of others.^18^

Despite these differences, Uniacke argues that DDE framing of dual use dilemmas aptly highlights the *moral responsibility* that scientists (and/or their funders) would have for harms they both *foresee* and *enable*.^19^ An implication, according to Uniacke, is that scientists engaged in such work (and presumably those funding it) have a moral obligation to ensure that risks associated with dual-use research they conduct (or fund) are minimized. It might also be argued that a version^20^ of the proportionality condition of DDE—i.e., that intended/expected benefits should outweigh foreseen harms (or risks)—should also apply to dual-use research.

### Principlism

#### Research Ethics

A number of popular approaches to bioethics appeal to principle-based frameworks. In the context of biomedical research involving human subjects, for example, the Belmont Report (DHHS 1979) argues that judgments about the ethics of research should be guided by the following overarching ethical principles:

- Respect for persons, which requires acknowledgement/respect of individual autonomy and protection of those with diminished autonomy. Application of this principle entails obligations regarding informed consent—i.e., “[human] subjects [of research], to the degree that they are capable, [should] be given the opportunity to choose what shall or shall not happen to them.”
- Beneficence, which requires that researchers “(1) do no harm and (2) maximize possible benefits and minimize possible harms.” Application of this principle entails “systematic assessment of risks and benefits”; that research involving human subjects “be justified on the basis of a favorable risk/benefit assessment” and/or “that risks to subjects be outweighed by the sum of both the anticipated benefit to the subject, if any, and the anticipated benefit to society in the form of knowledge to be gained from the research.”^21^
- Justice, which requires fair sharing of the benefits and burdens of research involving human subjects. Application of this principle requires “fair procedures and outcomes in the selection of research subjects,” i.e., those exposed to the risks of research.

Though explicitly designed to provide guidance regarding the ethical conduct of research involving human subjects in particular, it has been argued (Evans et al. 2015; Lipsitch and Galvani 2014) that the Belmont Report’s (and also the Nuremburg Code’s) beneficence requirements—e.g., that benefits outweigh risks, and that risks should be minimized—should also apply to GOFR. While this might be plausible, it might not be so obvious that Belmont’s informed consent requirement could or should straightforwardly apply to GOFR, because it would be impossible to seek/gain individual consent from all “capable” persons exposed to possible risks of GOFR. In the context of GOFR, it might be argued that Respect for Persons alternatively requires community consent and/or democratic processes.

#### Biomedical Ethics

A similar ethical framework developed and popularized by Tom Beauchamp and James Childress (2001) for biomedical ethics more generally appeals to a similar set of principles:

- Autonomy: individual autonomy should be respected/promoted.
- Non-maleficence: do not harm others.
- Beneficence: benefit others by protecting/promoting their well being.
- Justice: benefits and burdens should be shared fairly.

The Beauchamp and Childress framework largely mirrors that of the Belmont Report, but Beauchamp and Childress separate what is captured by the Belmont Report’s Beneficence principle into two separate principles (Non-maleficence and Beneficence). Beauchamp and Childress acknowledge that there may sometimes be conflict between their principles, and that a balance should, in such cases, be struck between them. If GOFR is expected to be especially beneficial (let’s assume, for example, the overall benefits for humanity outweigh the risks) but inevitably entails compromised autonomy (because it entails imposition of risk on individuals without their explicit consent) then the beneficence principle would conflict with the autonomy principle. The above discussion of Hansson likewise illustrates how beneficence might conceivably conflict with justice in the context of GOFR. The possibility of conflict between principles raises difficult questions about what would be a principled/legitimate way to strike a balance, or make trade-offs, between them (or the values they embody) in such cases.

#### Public Health Ethics

Recently developed frameworks for public health ethics are explicitly designed to address possible conflicts between liberty and utility that arise in cases where coercive (i.e., liberty-infringing) measures such as isolation and/or quarantine are necessary to protect/promote public health.

Among other things, public health ethics frameworks (Gostin 2006; Kass 2001; Selgelid 2009a; Upshur 2002) have posited that (1) liberty restriction in the name of public health protection should be based on evidence that the public health measure in question would in fact provide an effective means of public health protection, (2) the least restrictive (i.e., least liberty-infringing) alternative should be employed to achieve the public health goal in question, (3) extreme liberty-infringing methods such as isolation and quarantine should not be employed unless the consequences would otherwise be severe, (4) liberty-infringing interventions should be used in an equitable—i.e., non-discriminatory—manner and/or the bar for imposing such measures should be highest (with regard to the evidence required or the utility threatened) when those being considered for confinement are members of the worst off groups of society, (5) liberty-infringement should be minimally burdensome (e.g., so that those confined receive basic necessities and are made as comfortable as possible), (6) those whose liberty is violated should be compensated in return (7) implementation of liberty restrictions should involve due (legal) process, and those confined should have a right to appeal, and (8) relevant policy-making should (insofar as possible) be democratic and transparent.

Because imposition of risk on individuals could be conceived as a form of liberty-infringement, such principles (if legitimate) may have relatively straightforward application to the context of GOFR (aimed at public health protection/promotion). In any case, imposing risks on individuals without their (explicit) individual consent (in the case of GOFR aimed at public health protection/promotion) might be ethically problematic in a way that is similar^22^ to what is problematic about coercive public health measures. If this is correct, then it would not be surprising if analogous principles applied to the two kinds of cases.

In contrast with Beauchamp and Childress’ principlist framework, which is designed to highlight prima facie principles/values that should be satisfied/promoted when possible (rather than constituting necessary conditions), the public health principles outlined above are commonly framed as necessary conditions—each of which, it is argued, must be satisfied for liberty restriction aimed at public health promotion/protection to be ethically acceptable. Application of this kind of framework is not entirely straightforward, because it may often not be obvious whether any given principle is satisfied. With regard to (1), for example, how much and/or what kind of evidence would/should be needed?

## Towards an Ethical and Decision-Making Framework for GOFR (Funding) Policy-Making

In light of the preceding discussion of points raised in the GOFR ethics literature, limitations of RBA, and challenges to existing ethical and decision-making frameworks, the following framework might be considered appropriate for decision- and policy-making regarding the funding and conduct of GOFR. This framework is based on the idea that there is likely no (clearly correct) exact formula or algorithm that will solve hard questions about GOFR—and that judgments will inevitably need to be made. It thus highlights ethical desiderata that such judgments should be based upon, i.e., dimensions upon which policy makers (or decisions) could fare ethically better or worse. Because judgments will depend on numerous matters regarding which there is likely to be reasonable disagreement (i.e., matters that cannot be resolved by science and/or the discipline of ethics—e.g., questions about what is intrinsically valuable, the weightings that should be attributed to potentially conflicting values, appropriate levels of risk aversion, and/or appropriate risk-taking strategy), this framework suggests, among other things, that decision- and policy-making regarding the funding and conduct of GOFR should be as democratic as possible. Many of the hard ethical questions raised by GOFR, that is, should be resolved in a way that reflects the values and risk-taking strategies etc. of the people.

Because the US Department of Health and Human Services (DHHS) has determined that it will only fund GOFR where the expectation is that the study in question will be published (DHHS 2013), it should be noted at the outset that determination that any given study should not be published would entail that the study in question should not be funded by DHHS. Reaching such a determination, however, need not imply judgment that such a study should not take place at all, because studies not funded by DHHS might be funded privately and/or funded by other US government agencies to be conducted in a classified manner. It should also be noted that the decision not to fund any given study (even, ironically, in cases where such a decision is largely or partly based on concerns about publication dangers) is arguably less weighty than the decision to censor a study would be. Censorship involves direct interference with the scientific enterprise, academic freedom, and/or freedom of speech. While this does not necessarily mean that censorship would always be wrong it does mean that the grounds for censorship would need to be stronger than grounds for refraining from funding (a case of) GOFR—because refraining from funding (a case of) GOFR would not involve direct governmental interference with the scientific enterprise, academic freedom, or freedom of speech. The decision not to fund (a case of) GOFR might sometimes reflect the conclusion that (in light of an all-things-considered assessment of benefits and risks involved) there might be better uses of taxpayers’ money. Whether or not GOFR is involved, one should expect policy makers to consider possible risks/harms as well as benefits when making decisions about what research to fund (World Health Organization 2010). These preliminary remarks are by no means intended to downplay the potential value/importance or fundability of GOFR in general. As with non-GOFR studies, some (proposed) GOFR studies may be more socially valuable, and thus more worthy of funding, than others.

### Research Imperative

In cases where it is determined that GOFR (or publication thereof) may pose extraordinary risks to the public (or groups therein), the GOFR in question would be morally problematic. The ethical acceptability of GOFR (and publication thereof) thus partly depends on the extent to which there is an important reason to conduct (and publish) the GOFR in question. This principle appears to entail that, to be ethically acceptable, extraordinarily risky GOFR must address an important public health question. Conceived in a binary way (as in the previous sentence), however, a principle like this would be difficult to implement, because it raises arguably intractable questions about exactly how risky a study would need to be in order to be considered extraordinarily risky and exactly how important the research question would need to be in order for the research to satisfy the criterion in question.

Conceived as a scalar moral desideratum (rather than as a necessary condition/criterion that is either satisfied or not satisfied) the point of this principle is that, in cases where the research poses serious risks, its evaluation should *partly* be based on the importance of the research question it aims to address. Some research questions are obviously more important than others. The more important any given target research question, the more ethically acceptable it would be to fund/conduct/publish a study posing a given magnitude of risk (other things being equal). The less important any given research question would be, the less ethically acceptable it would be to fund/conduct/publish a study posing the same magnitude of risk (other things being equal). Generally speaking, furthermore, the riskier the research would be, the more important the research question would need to be in order for the research to be justified (other things being equal).

### Proportionality

The ethical acceptability of extraordinarily risky GOFR partly depends on the extent to which there is reasonable expectation that the research in question will (1) yield answers to the target public health question, and (2) ultimately result in public health benefits that outweigh risks involved. In any given case (depending on RBA findings) we might be more or less confident that the GOFR in question will actually satisfy these two conditions. Conceived as a scalar moral desideratum (rather than as a necessary condition/criterion that is either satisfied or not satisfied) the point of this principle is that, in cases where the research poses serious risks, its evaluation should *partly* be based on the level of confidence that (1) and (2) are satisfied. The greater confidence that (1) and (2) are satisfied, the greater the ethical acceptability of funding/conducting/publishing a study posing a given magnitude of risk—and vice versa. Other things being equal, furthermore, the greater the expected benefits of any given case of GOFR posing a given magnitude of risk, the more ethically acceptable it would be to fund/conduct/publish the study in question.

### Minimization of Risks

The idea that research risks should be minimized is a central tenet of human subjects research ethics. A call for risk minimization has likewise been widely appealed to in debates surrounding GOFR; and numerous ways in which risks related to GOFR might be minimized have been identified in the literature.

This kind of principle parallels the “least restrictive alternative” principle commonly appealed to in public health ethics. The latter holds that it would be unethical to employ more force/coercion than is necessary to achieve the public health goal in question—i.e., among alternative public measures that are otherwise ethically acceptable and equally effective, the measure involving the least force/coercion should be chosen. The least restrictive alternative principle in public health ethics, however, does not (necessarily) imply that a less restrictive measure should be preferred to a more restrictive measure if the former would entail compromised efficacy towards achieving the public health goal at issue.

In the context of GOFR, it is similarly plausible that (other things being equal) risky GOFR should not be pursued unless there is reason to believe that less risky kinds of research are unlikely or unable to *equally well* yield answers to the target public health question and thereby ultimately achieve public health benefits.^23^ As in the discussion of proportionality, in any given case we might have more or less confidence that a GOFR study is not more risky than other equally beneficial possible research alternatives,^24^ so the ethical acceptability of risky GOFR will be a function of the extent to which there is good reason for such confidence.

A further implication of the minimization of risk principle is that *when pursuing GOFR* we should minimize risks (at least insofar as possible without compromising expected benefits of the GOFR study in question). This raises the question of whether risks must be maximally minimized regardless of the (e.g., economic) costs and/or extent of risk reduction achieved—and/or what would be a “reasonable” cost to endure for marginal risk reduction. Again, if stated in binary terms, it is hard to imagine what a precise (plausible) minimization of risk principle should look like.

Conceived as a scalar moral desideratum (rather than as a necessary condition/criterion that is either satisfied or not satisfied) we might thus state this principle as follows: other things being equal, the ethical acceptability of (a given case of) GOFR is a function of the degree to which (1) there is confidence that no less risky forms of research would be equally beneficial (regarding the public health question/problem at issue) and (2) reasonable steps have been made to minimize risks of conducting the GOFR in question. This principle does not (necessarily) imply that a less risky study should be preferred to a more risky study if the former would be less beneficial.

### Manageability of Risks

Whether or not any given study should be funded/conducted/published partly depends on existing global “web of prevention” control measures in place rather than depending entirely on essential features of the GOFR study itself. Manageability of GOFR risks, like other relevant features considered above, is a matter of degree rather than either-or. Other things being equal, the more manageable the risks of (any given case of) GOFR (which partly depends on the strength of the background web of prevention in place), the more ethically acceptable the (case of) GOFR would be. Conversely, the more important/beneficial (any given case of) GOFR is expected to be, the more we should be willing to accept potentially unmanageable risks. It is also worth noting that severity of potentially unmanageable risks is also ethically relevant—because some potentially unmanageable risks might be less severe than others (and some potentially unmanageable risks might not be very severe at all).^25^

Here and in principles above (and below), a purpose of highlighting scalar dimensions of ethically relevant aspects of GOFR (i.e., highlighting that ethically relevant aspects of GOFR come in degrees rather than being either-or) is to reveal that: (1) appeal to either-or/binary criteria might not be sufficiently clear or action guiding (insistence that “risks of GOFR must be manageable or reasonably manageable”, for example, is arguably prohibitively vague); and (2) strict insistence on certain criteria might rule out too much. With regard to (2) we might imagine cases of especially important/beneficial GOFR—i.e., addressing crucial (and potentially otherwise unmanageable) risks—that it might be appropriate to pursue even if the GOFR in question poses nontrivial risks of unmanageability with nontrivial severity (though less unmanageability and less severity than the risks that the GOFR aims to address). (Some might think this, for example, about the controversial ferret H5N1 influenza studies.) As noted above, the acceptability of unmanageability of (any given case of) GOFR depends on the costs (in terms of forgone benefits) of refraining from (the case of) GOFR (in question).

### Justice

Justice requires fair sharing of research benefits and burdens. It would arguably be unjust if (1) GOFR risks fall upon some people (e.g., those living in countries with weak health care systems) more than others, (2) GOFR risks fall upon those who are unlikely to benefit from the research in question, and/or (3) individuals or groups suffer harms from GOFR without being compensated. As argued above in discussion of Hansson, though a perfectly equitable sharing of the risks and benefits of GOFR might be unrealistic given global political economics, it is reasonable to believe that the ethical acceptability of GOFR is a function of equity. Other things being equal, the more that is done to ensure equitable sharing of risks and benefits, the more ethically acceptable GOFR would be. Other things being equal, the less that is done to ensure equitable sharing of risks and benefits, the less ethically acceptable GOFR would be. Among other things, such a principle implies that the ethical acceptability of GOFR is a function of the degree to which (wealthy) countries conducting/funding GOFR (1) mitigate risks for those who are especially vulnerable (both domestically and internationally), (2) ensure wide availability of GOFR research benefits (both domestically and internationally), and (3) compensate those who suffer harm resulting from GOFR (both domestically and internationally).

### Good Governance: Democracy

The above discussion reveals numerous ways in which decision- and policy-making regarding GOFR turns on important, difficult questions—about ultimate values, value weightings, and risk-taking strategies, etc.—regarding which there will inevitably be reasonable disagreement. In a democracy, decision- and policy-making regarding GOFR should arguably (as far as possible) reflect the ultimate values, value weightings, and risk-taking strategies of the people (Kitcher 2001).

In addition to expert opinion (which is inevitably necessary), therefore, GOFR policy-making should involve systematic ongoing engagement with key stakeholders and the community at large—via processes of deliberative democracy^26^—in order to gain direct public input to decision-making and learn more about the ultimate values, value weightings, and risk-taking strategies that the public would like to see (and that thus should be) reflected/implemented by policy.

While individual informed consent to GOFR risks is obviously infeasible, community consent might address the Belmont Report’s (and other research ethics codes’) requirement of respect for persons—and deliberative democracy might be an ideal method for seeking community consent. Decision- and policy-making should, in any case, be as transparent as possible—because transparency plays a crucial role in democratic processes (Sen 1999).

In addition to being ethically important, democratic decision-making is important because democratic decision-making is necessary to maintain/improve public confidence and trust in both the scientific enterprise and government. Public trust and confidence are values that could be compromised (with adverse consequences) whether or not GOFR results in untoward outcomes. Such values may be compromised if the public is not satisfied that GOFR policy decisions adequately reflect the will (i.e., values, value weightings, risk-taking strategies, etc.) of the people and/or if it appears that GOFR policy entails unjust rights violations and/or is inequitable.

Susan Wolf and her colleagues (2009) and the Institute of Medicine (IOM) Committee on the Independent Review and Assessment of the NIH Recombinant DNA Advisory Committee (RAC) (IOM 2014) document the valuable role RAC has historically played in the promotion of public dialogue concerning ethical and social issues pertaining to gene transfer research involving human subjects. This has been achieved by its public review of especially challenging research protocols. The IOM RAC Report explicitly recommends considering possible establishment of a similar kind of venue for other emerging technologies raising important/difficult social and ethical issues. The IOM RAC Report suggests that such a venue might:

- Provide a public forum for the review and discussion of emerging areas of science
  - Include the capacity for a partnership to consult, inform, and educate institutional review boards (IRBs) and institutional biosafety committees (IBCs).
- Provide a venue to foster scientific and public awareness regarding emerging science in order to address concerns about clinical investigations and future societal implications.
- Integrate the capacity to surveil, aggregate, and analyze adverse events across related trials of emerging technologies.
- Perform an additional level of review of individual protocols that are identified by the NIH director, in consultation with one or more IRBs and IBCs, on the basis of exceptional issues raised (IOM 2014, pp. 6–7).

Though the IOM RAC Report is here explicitly referring to a venue concerned with *clinical research* involving new emerging technologies, analogous roles to many of those described above should arguably be filled by a relevant body in the context of GOFR,^27^ and more explicitly broadening the mandate of NSABB to fulfill such roles would be an obvious possibility.^28^ Whether or not *public review* of protocols, in particular, would be advisable in the context of GOFR is clearly open to question, because this could itself pose dual use dangers (via dissemination of potentially dangerous information and/or by promoting GOFR proliferation in worrisome kinds of cases).

### Evidence

The above discussion reveals that the ethical acceptability of GOFR depends on confidence regarding the (potentially unique) benefits and risks of conducting GOFR (in particular ways), how risks can be minimized,^29^ who might be likely to benefit or be harmed by the research in question, and the values and risk-taking strategies etc. of the people, which policy should aim to reflect. Confidence about such matters depends on the current state of knowledge, which can be improved via relevant empirical research. In some cases crucial ethical/policy decisions turn on answers to what are ultimately empirical questions. Answering such questions may thus be both scientifically and ethically important (Selgelid 2009a).

The RBA currently underway is a step in the direction of better-informed GOFR decision- and policy-making. Similar and/or relevant research (RBA and otherwise) concerning GOFR in general—and/or particular kinds of cases of GOFR—should continue in the future and receive relevant funding as necessary. The better informed any decision in favor of (or against) GOFR, the more ethically acceptable the conduct (or omission) of that GOFR would be.

Beyond processes of deliberative democracy, furthermore, carefully designed social research will be important for shedding light on people’s (reflectively held, as opposed to cognitively biased) ultimate values, value weightings, levels of risk aversion, and risk-taking strategies etc. that policy should aim to reflect.

Among other things, finally, the evidence principle entails careful ongoing monitoring of GOFR (e.g., with an eye to adverse events and compliance with safety protocols)—and it might sometimes require acquisition of and/or access to potentially classified intelligence information about the abilities, possessions, and intentions of malevolent actors or groups.

### International Outlook and Engagement

Because the risks and benefits of GOFR (can) affect the global community at large, the ethical acceptability of GOFR at least partly depends on the extent to which such research is accepted abroad. Decision- and policy-making regarding GOFR should arguably, insofar as is feasible, involve consultation, negotiation, coordination, and related forms of active engagement with other countries.

In its report *New Directions: The Ethics of Synthetic Biology and Emerging Technologies* The Presidential Commission for the Study of Bioethical Issues, for example, recommends

International Coordination and Dialogue … Recognizing that international coordination is essential for safety and security, the government should act to ensure ongoing dialogue about emerging technologies such synthetic biology. As part of [a] coordinated approach [the US Government] should continue and expand efforts to collaborate with international governments, the World Health Organization, and other appropriate parties, including international bioethics organizations, to promote ongoing dialogue about emerging technologies such as synthetic biology as the field progresses (Presidential Commission 2010, pp. 10).

This kind of recommendation is directly applicable to GOFR in particular (not least because GOFR will often itself involve synthetic biology).

## Conclusion

The ethical- and decision-making framework suggested above is based on the idea that there are numerous ethically relevant dimensions upon which any given case of GOFR can fare better or worse (as opposed to there being necessary conditions that are either satisfied or not satisfied, where all must be satisfied in order for a given case of GOFR to be considered ethically acceptable). Rather than drawing a sharp bright line between GOFR studies that are ethically acceptable and those that are ethically unacceptable, this framework is designed to indicate where any given study would fall on an ethical spectrum, where imaginable cases of GOFR might range from those that are most ethically acceptable (perhaps even ethically praiseworthy or ethically obligatory) (i.e., those that fare best with respect to all 8 dimensions), at one end of the spectrum, to those that are most ethically problematic or unacceptable (i.e., those that fare worst regarding all 8 dimensions, and thus clearly should not be funded/conducted), at the other. The aim should be that any GOFR pursued (and/or funded) should be as far as possible towards the former end of the spectrum.

One reason for resisting an approach based on necessary conditions is that the desiderata highlighted above involve ethically important factors that come in degrees, and it is hard to imagine that there are actually clear thresholds separating adequate from inadequate achievement of any given desideratum. In any given case of GOFR, our epistemic situation regarding achievement of any given desideratum will likewise be a matter of degree—i.e., there will be greater or lesser confidence regarding achievement level of each desideratum—and it is hard to imagine there being thresholds separating adequate from inadequate confidence. Another reason for resisting a framework based on necessary conditions is the intuition that compromised/suboptimal achievement of some desiderata might sometimes be compensated by high-level achievement of others.

Though the framework suggested here admittedly does not provide an algorithmic guide to action, it is doubtful that any clear algorithmic approach to evaluating GOFR would be justifiable or should be considered realistic or desirable. With regard to desirability, it is noteworthy that an algorithmic approach that merely aimed to separate ethically acceptable from ethically unacceptable cases of GOFR would fail to capture the degree to which any given study is acceptable or not. In cases of GOFR that fall at ends of the ethical spectrum, the framework suggested here (like an algorithmic approach) may give very clear guidance about what should be done. In cases of GOFR that fall in the middle/grey area, difficult judgments will need to be made, and, aside from the aim to achieve a democratic outcome (which should be an especially important desideratum), there might not always be clear right answers regarding whether a given case of GOFR should proceed (or be funded). Like risk-benefit assessment, ethics involves inevitable uncertainty.

## Footnotes

1 Whether or not ethics is their explicit main focus, most papers covered in this literature review were identified because they at least mention both ‘ethics’ and ‘gain-of-function research’ and thus turned up in systematic literature searches of relevant databases. Due to space constraints, the following does not aim to comprehensively cover literature concerned with dual-use life science research more generally—or even the controversial ferret H5N1 studies mentioned above, unless ‘gain-of-function research’ is explicitly mentioned—except insofar as the papers in question are directly relevant to risk-benefit analysis and/or the ethical- and decision-making framework parts of this paper. Much of the explicitly ethical literature concerned with dual-use life science research more generally, including the ferret H5N1 studies (prior to framing in terms of ‘gain-of-function research’), are largely concerned with the ethical responsibilities of scientists and/or issues of censorship which are beyond the scope of this paper, which focuses on the shift in debate (towards biosafety and risk-benefit analysis) that occurred with framing in terms of ‘gain-of-function research’.

2 This, of course, depends on the outcome of RBA. If, hypothetically, RBA demonstrated with a high degree of confidence that GOFR (or a certain case of GOFR, conducted under certain conditions) would be enormously beneficial in numerous ways without imposing major/significant risks—or if RBA demonstrated with a high degree of confidence that GOFR (or a certain case of GOFR, conducted under certain conditions) would be extremely risky in numerous ways without promoting major/significant benefits—then RBA might itself provide a clear guide to action. If things were this simple, however, GOFR would likely not be so controversial to begin with.

3 For discussion of “model risk”—i.e. “the risk that the model is inappropriate for the problem”—and ways in which this may be addressed, see the work of Peter Taylor (2012).

4 Given existing data regarding numbers of laboratory accidents and consequences thereof under various conditions (at least some) biosafety risks are being assessed quantitatively.

5 This, again, depends on RBA findings. If GOFR is determined to be especially beneficial (qualitatively speaking with regard to countermeasure development and/or quantitatively with regard to other benefits, if any are actually quantified) with no major/significant risks, then it might be safe to conclude that benefits outweigh risks.

6 With regard to economic benefits, money is a prototypical example of a merely instrumentally valuable good. Given complexities involved with GOFR RBA, it might be reasonable to consider number of lives saved or lost as a (simplifying) proxy measure for human well being (or public health) impact. Another possibility would be to quantify possible well being gains or losses in terms of DALYs (i.e., disability adjusted life years lost—which is a common measure of burden of disease).

7 According to utilitarian ethical theory well being is the only thing that is intrinsically valuable, and thus what policy should ultimately aim to maximize.

8 For related discussion of the value of security, and its relevance to health policy-making, see Jonathan Herington (2016).

9 Related/similar illustrations of this kind of approach to decision-making are provided by Thomas Douglas (2013) and David Resnik (2014). The example provided here is, for reasons discussed above and below, an over simplification of what actual choice situations regarding GOFR would be like; and the numbers used are not assumed to accurate or realistic.

10 Here, and in what follows, it is assumed that money (gained or lost) can be considered a proxy for utility (gained or lost)—just as number of lives saved or lost might be considered a reasonable (simplifying) proxy for utility in the case of GOFR.

11 This kind of objection to the expected utility maximization approach could arguably be addressed by accounting for risk-aversion—or the value of security—in the utility metric (i.e., rather than using number of lives lost or saved as a proxy for utility). Practical difficulties of the expected utility maximization approach (in the context of GOFR) discussed in what follows would nonetheless remain (and perhaps be exacerbated by more complicated utility metrics). Points (below) regarding the importance of democracy to value identification/weighting are likewise arguably applicable to expected utility maximization approaches employing more complicated utility metrics. For discussion of expected utility maximization approaches that aim to capture a plurality of potentially conflicting values, see Paul Weirich (2012).

12 Imagine, for example, that ordinary lottery tickets (with ordinary odds and payouts) cost thousands of dollars.

13 E.g., the “availability heuristic”—i.e., in light of past experience, some dangers more readily come to mind than others, so these become targets of what are actually disproportionate precautionary attitudes.

14 Cost-benefit analysis (CBA) (presumably, for Clarke) involves a decision procedure along the lines of expected utility maximization. CBA often involves conception of utilities in monetary terms in particular (Sunstein 2005), but it is not obvious that this is what Clarke has in mind. Clarke characterizes CBA as follows: “CBA involves attempting to determine the probability of benefits occurring, and the probability of costs being incurred, as well as determining the relative sizes of the benefits and costs of a particular course of action and balancing these. This calculation is compared with the relative balance of costs and benefits for alternative courses of action from which the option with the best overall balance, adjusting for probability of these occurring, is selected” (Clarke 2013, p. 224). That Resnik’s use of the precautionary principle involves conjunction with (rather than replacement of) cost-benefit thinking is revealed by his use of “reasonable” and his actual analysis. Resnik’s idea obviously is not that we must do whatever it takes to avoid dangers posed by dual-use research at any cost; his analysis reflects the idea that costs and benefits of precautionary action need to be considered (and the idea that balance between costs and benefits determine what is “reasonable”). Clarke might argue that Resnik’s precautionary approach sounds like a re-description of cost-benefit thinking rather than a replacement thereof.

15 The point being that the expected utility approach to decision- and policy-making ultimately aims at aggregate utility maximization without paying sufficient attention to (1) whether expected utility maximization entails rights violations or (2) whether or not utility is fairly distributed. Whether or not this is a fair criticism of expected utility maximization perhaps partly depends on how broadly “utility” is conceived—i.e., because disvalue of rights violations and/or inequality could arguably be factored into utility calculations in various ways. Hansson’s criticism of expected utility maximization might be fair, however, if utility is more narrowly conceived in terms of well being (or, as in examples offered above, the number of lives saved or lost).

16 Though Uniacke specifically considers application of DDE to scientists engaged in dual-use research where malevolent use of research findings is a foreseen possibility, much (but not all) of her analysis arguably also applies to (1) funders of research and (2) GOFR biosafety concerns. I add reference to funders in what follows.

17 Among other objections to DDE, critics commonly highlight difficulties distinguishing intended from merely foreseen consequences (which application of DDE requires).

18 Though Uniacke specifically focuses on concerns about malevolent use, possible laboratory accident (a foreseeable bad effect) in the case of GOFR could be directly produced either by the moral agent/scientist in question or others (e.g., in the case of proliferation).

19 Such moral responsibility would not necessarily entail moral blameworthiness. Moral agents in DDE scenarios are morally responsible for foreseen harms that they bring about—but they are arguably not morally blameworthy (if DDE conditions are met).

20 Taking probabilities into account.

21 Similar claims about the need for benefits to outweigh risk are embodied by other human research ethics frameworks (such as The Nuremburg Code) and US federal regulations.

22 i.e., doing potentially damaging things to people, and/or perhaps infringing upon their rights, in the aim to protect/protect public health.

23 It is likewise arguable, as suggested by Marc Lipsitch and colleagues, that risks might sometimes be minimized via pursuit of public health activities other than (GOFR) research—e.g., surveillance—that are equally (or more) beneficial than the GOFR under consideration. This point is implicitly addressed by the Research Imperative principle, because the importance of a research question is largely a function of the extent to which answering it is crucial to achievement of public health goals.

24 A similar point in the context of “least restrictive alternative” is made by Timothy Allen (unpublished).

25 Though arguably unmanageable, the weeds in my garden are tolerable.

26 That GOFR policy making should involve deliberative democracy has also been suggested by David Relman (Duprex et al. 2015).

27 Imperiale and Casadevall (2015, p. 5) have similarly suggested the possibility of “[c]reation of a national board to vet issues related to research with dangerous pathogens … [modeled] after the Recombinant DNA Advisory Committee. Such a board should have microbiological, infectious disease, biosafety, and ethical expertise, which, combined with access to national security information, would allow better assessments of biosafety and biosecurity issues.” For related proposals, see Selgelid (2007) and Miller and Selgelid (2008).

28 At least in cases where the roles in question are not already part of NSABB’s current mandate.

29 The need for additional biosafety research (and associated funding) in particular is also suggested by Evans and colleagues (2015). I here additionally have in mind research that helps determine whether GOFR (as opposed to other kinds of less risky research) is needed to answer key scientific questions.
